# Major niche transitions in Pooideae correlate with variation in photoperiodic flowering and evolution of CCT domain genes

**DOI:** 10.1093/jxb/erac149

**Published:** 2022-04-08

**Authors:** Siri Fjellheim, Darshan A Young, Martin Paliocha, Sylvia Sagen Johnsen, Marian Schubert, Jill C Preston

**Affiliations:** Department of Plant Sciences, Faculty of Biosciences, Norwegian University of Life Sciences, 1432 Ås, Norway; Department of Animal and Aquacultural Sciences, Faculty of Biosciences, Norwegian University of Life Sciences, 1432 Ås, Norway; Department of Plant Sciences, Faculty of Biosciences, Norwegian University of Life Sciences, 1432 Ås, Norway; Department of Plant Sciences, Faculty of Biosciences, Norwegian University of Life Sciences, 1432 Ås, Norway; Department of Plant Sciences, Faculty of Biosciences, Norwegian University of Life Sciences, 1432 Ås, Norway; Department of Plant Biology, The University of Vermont, Burlington, VT 05405, USA

**Keywords:** CCT domain genes, *CONSTANS*-like genes, flowering, grasses, photoperiod, Pooideae, *VRN2*

## Abstract

The external cues that trigger timely flowering vary greatly across tropical and temperate plant taxa, the latter relying on predictable seasonal fluctuations in temperature and photoperiod. In the grass family (Poaceae) for example, species of the subfamily Pooideae have become specialists of the northern temperate hemisphere, generating the hypothesis that their progenitor evolved a flowering response to long days from a short-day or day-neutral ancestor. Sampling across the Pooideae, we found support for this hypothesis, and identified several secondary shifts to day-neutral flowering and one to short-day flowering in a tropical highland clade. To explain the proximate mechanisms for the secondary transition back to short-day-regulated flowering, we investigated the expression of CCT domain genes, some of which are known to repress flowering in cereal grasses under specific photoperiods. We found a shift in *CONSTANS 1* and *CONSTANS 9* expression that coincides with the derived short-day photoperiodism of our exemplar species *Nassella pubiflora*. This sets up the testable hypothesis that *trans*- or *cis*-regulatory elements of these CCT domain genes were the targets of selection for major niche shifts in Pooideae grasses.

## Teaser

Long-day flowering evolved early in the grass subfamily Pooideae, with later, novel evolution of short-day flowering in Stipeae, probably associated with evolution of CCT domain gene expression.

## Introduction

The ability of plants to coordinate flowering with favorable environmental conditions results in optimization of reproductive fitness through increased seed set and survival (Greenup *et al.*, 2009). The exact timing of flowering is determined by several different external (e.g. photoperiod and temperature) and internal (e.g. age and hormone) signals that are integrated at the shoot apical meristem (SAM) throughout the lifetime of the plant. In the non-equatorial tropics, shortening photoperiods signal that the rainy season or monsoon is coming to an end, resulting in flowering of short-day grasses (Poaceae) such as rice (*Oryza sativa*) and maize (*Zea mays*) at the end of the greening period, prior to the extreme heat of summer (Naranjo *et al.*, 2014; Mascheretti *et al.*, 2015; Preston and Fjellheim, 2020). In contrast, lengthening photoperiods during the impending warm season of temperate regions trigger flowering in long-day plants such as the grasses wheat (*Triticum aestivum*) and barley (*Hordeum vulgare*), circumventing the negative effects of winter freezing (Nishida *et al.*, 2013; Chen *et al.*, 2014). Photoperiodicity in flowering is thus a good predictor of current plant distributions (Zhang *et al.*, 2015; Preston and Fjellheim, 2020), but the evolutionary genetic basis of switches between long- and short-day responses is not well understood.

Similar to angiosperms as a whole (Hochuli and Feist-Burkhardt, 2013; Mannion *et al.*, 2014), the grass family evolved when the terrestrial Earth was largely tropical (Burke *et al.*, 2016; Gallaher *et al.*, 2019; Schubert *et al.*, 2019a), suggesting that the ancestor would either have flowered under short days or been daylength neutral (Preston and Fjellheim, 2020). Indeed, of the ~12 000 extant grass species (Soreng *et al.*, 2015), the majority remain in the tropics, with only a couple of major subfamilies—Danthonioideae and Pooideae—dominating southern and northern temperate regions, respectively (Edwards and Smith, 2010; Visser *et al.*, 2014; Schubert *et al.*, 2020). Evidence suggests that the ability of an early Pooideae ancestor to respond to inductive photoperiods was contingent upon receiving a prolonged period of winter cold (vernalization) (McKeown *et al.*, 2016), although data also suggest later modifications to this ancestral vernalization pathway (Woods *et al.*, 2016). It is further hypothesized that the last common ancestor of Pooideae evolved from a daylength-neutral/short- to a long-day plant, the mechanisms underlying which are unknown (Preston and Fjellheim, 2020).

Comparative analyses across both long- and short-day angiosperms have revealed remarkable conservation in the photoperiod flowering pathway, suggesting that flowering in response to different daylengths evolved through fine-tuning of a shared ancestral pathway (Andrés and Coupland, 2012; Matsubara *et al.*, 2014). Central in this pathway is the florigen FLOWERING LOCUS T (FT). FT and related proteins act as universal signals to integrate flowering pathways and promote reproduction. Crucial for perception of photoperiod are various light receptors, one of which is *PHYTOCHROME C* (*PHYC*). *PHYC* is a weak floral repressor in short days in rice (Takano *et al.*, 2005), whereas it promotes flowering under long days in barley and *Brachypodium distachyon* (Nishida *et al.*, 2013; Woods *et al.*, 2014). Another gene family that has been implicated in fine-tuning flowering is the CCT [CO, CO-LIKE, and TIMING OF CAB EXPRESSION 1 (Robson *et al.*, 2001)] domain gene family of transcription factors, with nine members in long-day barley (Pooideae) and 16 members in short-day rice (Oryzoideae) (Griffiths *et al.*, 2003; Song *et al.*, 2015). Examples of CCT domain-containing genes implicated in intraspecific variation in flowering responses are barley *PHOTOPERIOD 1* (*PPD1*) and its ortholog *PSEUDORESPONSEREGULATOR 37* (*PRR37*) in rice, barley *CO1* and *CO2* and their ortholog *HEADING DATE 1* (*Hd1*) in rice, *CO9*, and barley *VERNALIZATION 2* (*VRN2*) and ortholog *Grain number, plant height, and heading date 7* (*Ghd7* or *OsI*) in rice (Komiya *et al.*, 2008; Xue *et al.*, 2008; Stracke *et al.*, 2009; Takahashi *et al.*, 2009; Lu *et al.*, 2012; Koo *et al.*, 2013; Wei *et al.*, 2014; Zhang *et al.*, 2015; McKeown *et al.*, 2016; Zheng *et al.*, 2016; Zhang *et al.*, 2017; Shaw *et al.*, 2020).

Like its *CO* ortholog in *Arabidopsis thaliana* (Brassicaceae), *CO1* in barley and wheat is up-regulated in the afternoon by the *PHYTOCHROME A* and *B* (*PHYA/B*)-mediated circadian clock under both long- and short-day conditions (Campoli *et al.*, 2012; Mulki and von Korff, 2016). In *A. thaliana*, photoperiod regulation through *CO* occurs at the protein level in the presence of light-induced stabilizing proteins, resulting in the up-regulation of *FT* to induce flowering only under long days (Yanovsky and Kay, 2002; Valverde *et al.*, 2004; Hayama *et al.*, 2017). Although it has not been confirmed that similar light-induced protein stabilization exists for CO1, genetic evidence from barley and wheat cultivars with non-functional *PPD1* alleles has shown that this protein also promotes flowering under long-day conditions, concomitant with peak expression in the light (Campoli *et al.*, 2012; Mulki and von Korff, 2016; Shaw *et al.*, 2020). On the other hand, in the presence of functional *PPD1* and *VRN2* alleles, at least wheat *CO1* is converted to a mild floral repressor under long days to prevent precocious pre-winter flowering, probably as a result of protein–protein interactions between PPD1, CO1, CO2, and possibly VRN2 (Shaw *et al.*, 2020).

In rice, the CO1 ortholog Hd1 is also assumed to be regulated by light- and dark-dependent proteins, and also forms an Hd1/CO1–PRR37/PPD1–Ghd7/VRN2 protein complex under long days to repress flowering via repression of *Early heading date 1* (*Ehd1*) and hence *FT/Hd3a* (Griffiths *et al.*, 2003; Xue *et al.*, 2008; Zhang *et al.*, 2015; Fujino *et al.*, 2019). Together with the fact that rice *Hd1* and wheat *CO2* promote and repress flowering under short days, respectively, these data support a role for changing *CO*-like protein interactions in transitions between short-day, day-neutral, and long-day flowering photoperiodism (Kitagawa *et al.*, 2012; Song *et al.*, 2015; Mulki and von Korff, 2016).

In addition to positively and negatively regulating *FT* (also named *VRN3*; Yan *et al.*, 2006) in barley and wheat, CO1 and CO2 are involved in a regulatory feedback loop with VRN2 (Mulki and von Korff, 2016). *VRN2* is a monocot-specific repressor of flowering that is negatively regulated by vernalization in the large ‘core’ Pooideae clade, comprising species such as wheat, ryegrasses (*Lolium* sp.), and oats (*Avena* sp.). However, *VRN2* is not down-regulated in response to cold in other ‘non-core’ Pooideae clades, including vernalization-responsive *B. distachyon* (Woods *et al.*, 2016). Under long days of the early autumn, winter barley *VRN2* is strongly up-regulated in leaves by the action of CO1, CO2, and PPD1 (Distelfeld *et al.*, 2009). Overexpression of *CO1* and *CO2* in spring barley results in up-regulation of *VRN2*, leading to delayed flowering in both long and short days (Mulki and von Korff, 2016). In turn, VRN2 negatively regulates *CO1/2* and *PPD1*, thereby dampening its own expression (Mulki and von Korff, 2016). As winter approaches, low-temperature-induced expression of the flowering promoter *VERNALIZATION 1* (*VRN1*; Oliver *et al.*, 2009) results in the gradual repression of *VRN2*, and a concomitant increase in *FT*, partly mediated by CO1/2 and PPD1 (Song *et al.*, 2015; Mulki and von Korff, 2016).

*CO9* is a grass-specific paralog of *VRN2/Ghd7* (Woods *et al.*, 2016), and overexpression in rice suggests that it acts as a floral repressor (Kikuchi *et al.*, 2012). Based on expression and functional analyses, this occurs under both short and long days, where transcript abundance peaks early after dawn (Kikuchi *et al.*, 2012). Since barley *VRN2* is expressed at its highest level towards the end of the light period (Trevaskis *et al.*, 2006), and rice *Ghd7* is expressed at high levels throughout the light period (Xue *et al.*, 2008), these data suggest evolution of *VRN2/CO9* genes in terms of both photoperiodic and circadian regulation following both duplication and speciation events.

Here, we reconstruct the evolution of photoperiodic flowering in Pooideae to test the hypothesis that flowering in response to long days evolved early in the subfamily and hence facilitated a range shift into northern temperate regions. We show that ancestral Pooideae was probably long day responsive, and that a secondary transition back to tropical climates was coincident with a shift back to short-day flowering. To determine if this derived short-day responsiveness can be explained by changes in the (co-)expression of CCT domain genes, we assess relative transcript levels for long- and short-day light–dark cycles across time in exemplar long- and short-day flowering species.

## Materials and methods

### Plant growth and experimental conditions

Forty-seven Pooideae species (13 core and 34 non-core) and the outgroup *Ehrharta calycina* from subfamily Oryzoideae (Supplementary Table S1) were selected to represent phylogenetic and geographic diversity across Pooideae. The plants were grown under different treatment conditions to score for long-day, short-day, or day-neutral flowering. Fifteen plants were grown per treatment. All seeds were stratified in moist soil (Gartnerjord, Tjerbo Torvfabrikk AS, Rakkestad, Norway) in complete darkness for 6 d, first under 4 °C for 5 d, followed by 1 d at room temperature. Seeds were then transferred to an open greenhouse in long days (16 h light:8 h dark) at 17 °C and grown for 4 weeks before the plants were randomized and assigned to one of four treatments: 17 °C short days (8 h light:16 h dark), 17 °C long days, 4 °C short days, or 4 °C long days for 12 weeks. We included vernalization in two of the treatments to see the effect of photoperiod even in vernalization-responsive species. All short- and long-day-grown plants were then maintained in short or long days, respectively, at 17 °C until flowering (calculated as days to heading) or termination of the experiment at 200 d. The experiment was repeated following the same conditions, except for reduction of the vernalization period to 8 weeks, switching the upper temperature to 20 °C, and termination of the experiment at 120 d. Light intensity under vernalization was 50 ± 5 µmol m^−2^ s^−1^, and for all other conditions it was 150 ± 10 µmol m^−2^ s^−1^. Light used in the experiment was produced by HQI lightning systems (LU400/XO/T/40 Philips Osram, General Electric, Hungary) giving a red/far-red ratio of 1.8 ± 0.2. Plants were watered and fertilized (water containing 4% Yara Kristalon Indigo and 3% YaraTera Calcium Nitrate, Yara Norway AS), adjusted to an electron conductivity of 1.5 as needed, and moved to a new position twice a week within the chamber.

To investigate more closely molecular responses to different photoperiods, two non-core Pooideae species in tribe Stipeae, long-day *Oloptum miliaceum* (USDA GRIN PI207772) and short-day *Nassella pubiflora* (USDA GRIN PI478575), and the long-day flowering Meliceae species *Melica ciliata* (Millennium Seed Bank 31675) were chosen for a follow up-experiment based on results from the first growth experiment. *Ehrharta calycina* (USDA GRIN PI284803 and PI578674) from the subfamily Oryzoideae was included as an outgroup. None of these species had an absolute vernalization requirement. Growth experiments were performed in two Conviron CMP6010 (Conviron, Winnipeg, Canada) growth chambers. Approximately 160 seeds of each of *M. ciliata*, *N. pubiflora,* and *O. miliaceum*, and 88 seeds of *E. calycina* were sown on moist filter paper and stratified under darkness for 4 d at 4 °C followed by 1 d at room temperature. Seeds were then planted in Metro-Mix 380, grown under long days at 20 °C for 4 weeks, and randomly assigned either to a long-day 20 °C or short-day 20 °C treatment until flowering, death, or termination of the experiment. For each plant, days to heading, number of leaves on the main stem at flowering, and tillers at flowering were recorded. The top fully expanded leaf of at least three plants without repeated measures were sampled at 2, 16, and 30 d after the initial 4 weeks growth under long days at 2, 8, 14, and 20 h post-dawn (ZT).

### DNA extraction and sequencing

Genomic DNA was extracted from leaf material using the DNeasy Plant MiniKit (Qiagen, Valencia, CA, USA), following the manufacturer’s protocol. We obtained sequences for three DNA plastid regions *matK*, *ndhF*, and *rbcL*, using custom Pooideae-specific primers (Schubert *et al.*, 2019a; Supplementary Table S2). PCR was performed on a Tetrad 2 Thermal Cycler (Bio-Rad, Hercules, CA, USA) and a Mastercycler ep Gradient Thermal Cycler (Eppendorf, Hamburg, Germany) using JumpStart REDTaq ReadyMix (Sigma-Aldrich, St. Louis, MO, USA) and standard conditions with 58 °C annealing and 2 min extension. PCR products were Sanger-sequenced in both directions using the same primers as for PCR. Chromatograms and sequences were inspected in BioEdit (Hall, 1999), and automatic alignments generated with manual adjustments (Supplementary Datasets S1–S3).

### Ancestral state reconstruction of flowering responses

Phylogenetic trees were generated for the concatenated *matK*, *ndhF*, and *rbcL* chloroplast dataset in MrBayes on XSEDE (Miller *et al.*, 2010) implemented through the CIPRES Science Gateway v.3.3. The dataset was partitioned by gene, rooted with sequences from maize (*Zea mays* ssp. *mays*), and run twice for 10 million generations sampling every 1000 generations, with four chains, 25% burn-in, and other default parameters. The consensus tree was visualized in FigTree v1.4.3 (http://tree.bio.ed.ac.uk/software/figtree/) and edited in Adobe Illustrator CS6. To account for uncertainty in topology prior to ancestral state reconstruction, 200 rooted trees with branch lengths were collated from the two independent runs as input for BayesTraits v2 (Pagel *et al.*, 2004; Pagel and Meade, 2006). BayesTraits was run using the Multistate function, and a one-rate/symmetrical model was chosen based on results of stepping-stone estimation comparing symmetrical and asymmetrical state transition models. Markov chain Monte Carlo (MCMC) analyses were run with 10 million generations, sampling every 1000th generation, with a burn-in of 25%. Trait states for all internal nodes in the Bayesian consensus tree were inferred by calculating the means of posterior probability distributions for each node.

### Scanning electron microscopy

To document if differences in time of transition reflect the flowering phenotype, we chose a representative subset of our focal species to investigate this at the level of SAM development under different photoperiods. We documented the developmental stage of *O. miliaceum*, *N. pubiflora*, and *E. calycina* SAMs across time points and treatments by dissecting meristems and subjecting them to SEM. At 2, 16, 27, and 41 d after onset of treatment, three SAMs from each species were fixed in formalin acetic acid (FAA) (50% ethanol, 5% glacial acetic acid, 10% of 37% formaldehyde) solution for 8–12 h. Following this, meristems were progressively transferred in five steps from 50% to 100% ethanol before critical point drying. Meristems were mounted on stubs, sputter coated with argon, and photographed using a JEOL 6060 SEM with an accelerating voltage of 25 kV.

### RNA extraction, cDNA synthesis, and quantitative PCR

Leaves of *M. ciliata*, *O. miliaceum*, *N. pubiflora*, and *E. calycina* were flash-frozen in liquid nitrogen, stored at –80 °C, and later macerated for RNA extraction using TriReagent (Ambion, Thermo Fisher Scientific, Waltham, MA, USA) followed by removal of DNA by DNase treatment with the TURBO DNA-free kit (Ambion). cDNA was then synthesized from 500 ng of RNA using the iScript cDNA synthesis kit (Bio-Rad, Hercules, CA, USA). All procedures followed the manufacturer’s instructions.

A *CO9* ortholog from *E. calycina* and *VRN2* orthologs from *N. pubiflora* and *O. miliaceum* were amplified in a standard PCR with cDNA pooled across time points and treatments for each species using previously published (Woods *et al.*, 2016), as well as newly designed, primers (see Supplementary Table S2). Amplicons were ligated into pGEM-T (Promega, Madison, WI, USA), plasmids used to transform competent DH5α *Escherichia. coli* cells, and ~10 clones were sequenced per amplicon by the Advanced Genomes Technology Core at The University of Vermont. *VRN2* from *M. ciliata* has previously been published (Woods *et al.*, 2016). To identify orthologs of *CO9* from *O. miliaceum*, *N. pubiflora*, and *M. ciliata*, as well as orthologs of *PPD1*, *CO1*, and *PHYC* from all Pooideae species, we generated transcriptomes for each species from leaves sampled in both conditions throughout a 24 h cycle. Briefly, leaves were flash-frozen in liquid nitrogen, and total RNA was extracted from homogenized tissue using the RNeasy Plant Mini Kit (Qiagen) including purification using the Invitrogen TURBO DNA-free kit (Thermo Fisher Scientific). Sequencing libraries with an insert size of 350 bp were constructed with the TruSeq Stranded mRNA Library Prep kit (Illumina, San Diego, CA, USA). Library preparation and paired-end sequencing was carried out by the Norwegian Sequencing Centre (NSC) at the University of Oslo on an Illumina HiSeq 4000 System (Illumina) with 150 bp reads. Read trimming and quality assessment of the transcriptomes followed Schubert *et al.* (2019b). The target sequences were identified through a BLAST search against the transcriptomes from the respective species using verified sequences from *H. vulgare* as queries (Supplementary Datasets S4–S7). 

Nucleotide sequences of *PHYC*, *PPD1*, *CO1/CO2/Hd1*, *VRN2/Ghd7*, or *CO9* were identified in model grass species through BLAST searches using verified sequences from *H. vulgare* as queries (see Supplementary Datasets S4–S7). To verify orthology, new sequences from our focal species were aligned with sequences of model species using MAFFT (Katoh and Standley, 2013) followed by manual adjustments, and maximum likelihood phylogenetic analysis using PHYML through NGPhylogeny.fr using default parameters and 500 bootstrap replicates (Dereeper *et al.*, 2008; Lemoine *et al.*, 2019).

For each target gene and focal species, we designed primers for quantitative reverse transcription–PCR (RT–qPCR) (Supplementary Table S2). Primers for *VRN3* and the housekeeping genes *UBIQUITIN 5* (*UBQ5*) and *ELONGATION FACTOR 1α* (*EF1α*) were either previously published (Ream *et al.*, 2014) or designed based on conserved regions in alignments of *Lolium perenne*, wheat, and *Oryza brachyantha* or rice, whereas *VRN2* primers were constructed based on previously published alignments (McKeown *et al.*, 2016). All new primers were designed using Primer3 (Rozen and Skaletsky, 2000), and the amplification efficiency of each primer pair was determined using a dilution series as previously described (Scoville *et al.*, 2011). To quantify relative gene expression, target gene critical threshold c(T) values were normalized against the geometric mean of the two housekeeping genes after correction for primer efficiency with three technical and at least three biological replicates.

### Western blot

An alignment was made of translated transcript sequences of *CO9* and *VRN2* from *N. pubiflora*, *O. miliaceum*, and *M. ciliata* as well as a selection of other grass species (Supplementary Dataset S8). Polyclonal antibodies were constructed for *N. pubiflora* and *O. miliaceum* (antigenic peptide sequence RRGMRCGVADLNRGC) and *M. ciliata* (a mix of the antigenic peptide sequences AGRRCGVAADLNLRC and VDQQEPAVIGGGGAC) to avoid cross-reactivity with VRN2. Leaf tissue was sampled from three biological replicates of each species subjected to long or short days at ZT2, 8, 14, and 20 one week after start of treatment, as previously described. Approximately 100 mg of tissue was ground in liquid nitrogen using a mortar and pestle, before adding 200 µl of DTE extraction buffer [3 mM DTT, 20 mM sucrose, 3 mM Na_2_CO_3_, 0.5% SDS, 1 mM EDTA, and 1:100 v/v of protease inhibitor cocktail (Sigma)]. Each sample was mixed briefly by vortexing, sonicated for 2.5 min (5 s on, 5 s off for a total of 5 min), and centrifuged at 12 000 *g* for 20 min at 4 °C. The supernatant was then transferred to a fresh tube and centrifuged for another 15 min. A 50 µl aliquot of the supernatant was precipitated using 500 µl of 10% trichloroacetic acid (TCA), and centrifuged at maximum speed (20 000 *g*) for 10 min at 4 °C. The liquid was removed, and the pellet left to air-dry before being dissolved in 0.1% NaOH. The concentration of protein extract was measured using a Qubit protein assay kit after adding 1:1 volume of 2× Laemmli sample buffer containing 5% β-mercaptoethanol. Three technical replicates of protein extract were incubated at 75 °C for 10 min, put briefly on ice, and centrifuged at 12 000 *g* for 1 min at 4 °C.

A 25 µg aliquot of protein was applied to a 12% Mini-PROTEAN® TGX Stain-Free™ Precast Gel, using 3 µl of Precision Plus Protein Unstained Standard as a marker. The gel was run at 200 V for 40-45 min in 1× Tris/Glycine/SDS buffer (Bio-Rad), UV-activated for 1 min using GelDoc Stain Free gel application, and the proteins blotted onto a 0.2 µm polyvinylidene difluoride (PVDF) membrane using the Trans-Blot Turbo Mini 0.2 µm PVDF Transfer Packs (Bio-Rad) and Trans-Blot® Turbo™ Transfer System (Bio-Rad). The Turbo program was set at 25 V and 2.5 mA for 3 min, and the membrane was analyzed using GelDoc Stain Free Blot application for loading control and normalization as per the manufacturer’s instructions. After being left to air-dry, the membrane was activated for 3 min with methanol and blocked in 2% dry milk solution in 1× TBS-T (500 mM NaCl, 20 mM Tris–HCl pH 7.5) for 1 h at room temperature. After washing twice for 10 min in TBS-T, the membrane was incubated with primary antibody diluted in blocking solution at 4 °C overnight. Dilutions were 0.25 µg ml^–1^ for *N. pubiflora* and 0.5 µg ml^–1^ for *O. miliaceum*. Following this, membranes were washed for 6 × 10 min in TBS-T followed by incubation for 1 h at room temperature with a 1:1000 dilution of mouse anti-rabbit horseradish peroxidase (HRP)-conjugated secondary antibody (SC-2357 Santa Cruz Biotechnology, Dallas, TX, USA). Subsequently, the membrane was washed 6 × 10 min in TBS-T and the signal was developed using Clarity™ Western ECL Substrate (Bio-Rad). After visualizing the signal using the GelDoc Chemi application, quantitation and analysis were performed using the Image Lab 6 software.

### Statistical analyses

To capture both qualitative and quantitative variation in flowering behavior across species, we calculated both the proportion of individuals flowering per treatment and absolute dates to heading per treatment. In cases where flowering consistently occurred in the absence of vernalization, we used non-vernalized long- and short-day-treated plants to calculate the photoperiod response. However, when plants had an absolute requirement for vernalization to flower, we used vernalized long- and short-day-treated plants to calculate the photoperiod response. We classified species as long day responsive if the proportion of individuals flowering was significantly more (*P<*0.05 as determined by a χ^2^ test), and/or days to heading was significantly less (*P<*0.05 determined by a two-tailed *t*-test) in long as compared with short days, and vice versa for short-day-responsive species.

For the relative gene expression data, two-way ANOVAs were performed with expression of *PHYC*, *PPD1*, *CO1*, *VRN2*, and *CO9* as dependent variables, and treatment and ZT time as independent variables. We removed the effect of sampling day (samples were taken at days 2, 16, and 30 after onset of treatment) by centering and standardizing expression data for all days using the ‘scale’ and ‘center’ functions in R. This was repeated for all genes and species, except for *VRN3* that is expected to increase in expression only after receiving several upstream inductive signals; in this case, expression was analyzed over the three sampling days separately. Analyses were done using both raw and transformed data, and analyses where the residuals best fitted a normal distribution were chosen for further interpretation. To investigate the effect of photoperiod on expression at specific time points, we performed post-hoc contrasts for all species, genes, and time points. All ANOVAs and post-hoc tests were carried out in R (RCoreTeam, 2016) using the stats and emmeans (Lenth, 2021) packages.

Each western blot was run with one complete set of samples from both treatments from one species, with three technical replicates. Three biological replicates were run per species. One of the replicates of *M. ciliata* produced smeared bands and we were unable to quantify protein abundance. As values cannot be compared directly across different blots, we removed the effect of blotting gels by centering and standardizing the protein expression data per blot using the ‘scale’ and ‘center’ functions in R, before averaging over technical replicates, and the biological replicates for each time points in each treatment. Graphs of mRNA and protein abundance were plotted in R (RCoreTeam, 2016), using packages ggplot2 (Wickham, 2016), tidyverse (Wickham *et al.*, 2019), ggalt (Rudis *et al.*, 2017), and patchwork (Pedersen, 2019). We visually inspected the resulting graphs to find the diurnal expression pattern.

## Results

### Long-day flowering evolved early in Pooideae

**Fig. 1.**
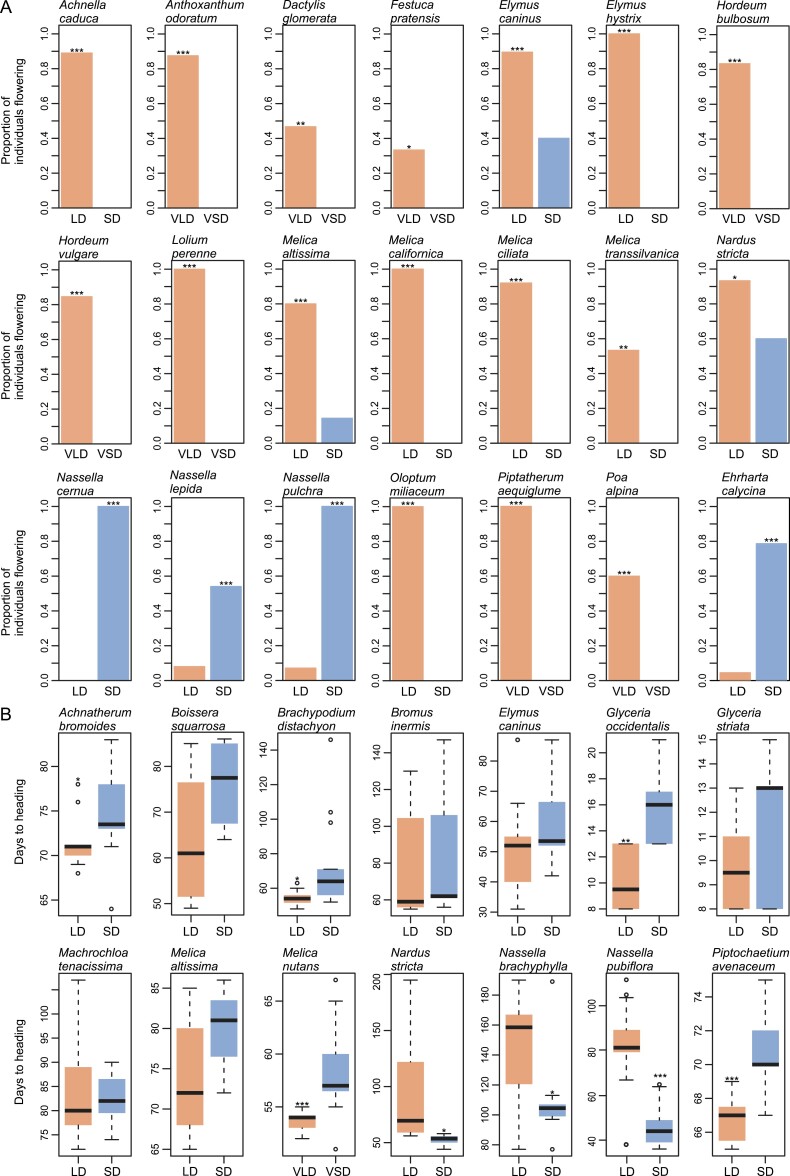
Flowering behavior for 31 of the 47 tested Pooideae species as well as *Ehrharta calycina* of the Oryzoideae. Comparisons were made between either long- or short-day treated plants (LD/SD) or between vernalized plants followed by long- or short-day treatments (VLD/VSD). (A) Barplots of proportion of individuals flowering under different photoperiods. (B) Boxplots of days to heading under different photoperiods. Three species are included in both (A) and (B) as they flowered in both compared treatments, but in inadequate numbers for *t*-tests (*Melica altissima*) or results were conflicting between comparisons of proportion of plants flowering and heading dates (*Elymus caninus* and *Nardus stricta*). *Nassella pulchra* and *Nassella lepida* flowered with only one individual in one of the treatments, and plots for heading date are not shown. The remaining 18 species failed to flower consistently enough to score. *P>0.05, **P>0.005, ***P>0.001. Red color indicates long-day treatment and blue color indicates short-day treatment.

Of the 47 Pooideae species tested for flowering responses to different photoperiods, we characterized 21 as long day responsive, five as short day responsive, and five as day neutral. For the remaining species, five (*Diarrhena obovata*, *Duthiea brachypodium*, *Hesperostipa spartea*, *Nassella neesiana*, and *Schizachne purpurascens*) failed to give a statistically clear response due to too few individuals flowering, and 11 species were completely non-flowering (*Ampelodesmos mauretanicus*, *Brachypodium pinnatum*, *Brachypodium sylvaticum*, *Diarrhena americana*, *Helictrotrichon hookeri*, *Helictotrichon pubescens*, *Lygeum spartum*, *Phaenosperma globosa*, *Stipa barbata*, *Stipa lagascae*, and *Stipa pennata*; Fig. 1). Twenty-two species flowered in adequate numbers without vernalization, five of which were identified as short day responsive, either because they flowered significantly faster (*N. pubiflora* and *Nassella brachyphylla*, *t*-test, *P<*0.05) or because significantly more individuals flowered (*Nassella cernua*, *Nassella lepida*, and *Nassella pulchra*, χ^2^ test, *P<*0.05) in short than in long days. The five species identified as day neutral either showed no significant difference in flowering time between photoperiodic treatments (*Glyceria striata*, *Macrochloa tenacissima*, *Bromus inermis*, and *Boissera squarrosa*, *t*-test, *P*>0.05) or produced conflicting results between different treatments in flowering time and frequency (*Nardus stricta*). Twelve species were identified as long day responsive in the absence of vernalization due to them flowering faster (*Glyceria occidentalis*, *Achnatherum bromoides*, *Piptochaetium avenaceum*, and *Achnella caduca*, *t*-test, *P<*0.05), or with significantly more individuals flowering (*Brachypodium distachyon*, *Melica altissima*, *Melica californica*, *M. ciliata*, *Melica transsilvanica*, *O. miliaceum*, *Elymus caninus*, and *Elymus hystrix*, χ^2^ test, *P<*0.05) in long versus short days.

Nine species (*Melica nutans*, *Festuca pratensis*, *Poa alpina*, *Dactylis glomerata*, *Anthoxanthum odoratum*, *Lolium perenne*, *Piptatherum aequiglume*, *Hordeum bulbosum*, and *Hordeum vulgare*) flowered in adequate numbers only after vernalization. Of these, only *M. nutans* flowered in response to both photoperiods and was scored as long day responsive because flowering was faster in long compared with short days (*t*-test, *P<*0.05). All other Pooideae species were evaluated as long day responsive as significantly more individuals flowered in long than in short days (χ^2^ test, *P<*0.05). As predicted, the outgroup species *E. calycina* (Oryzoideae) was classified as short day responsive as it flowered significantly more in short versus long days without vernalization (*P*<0.05).

**Fig. 2.**
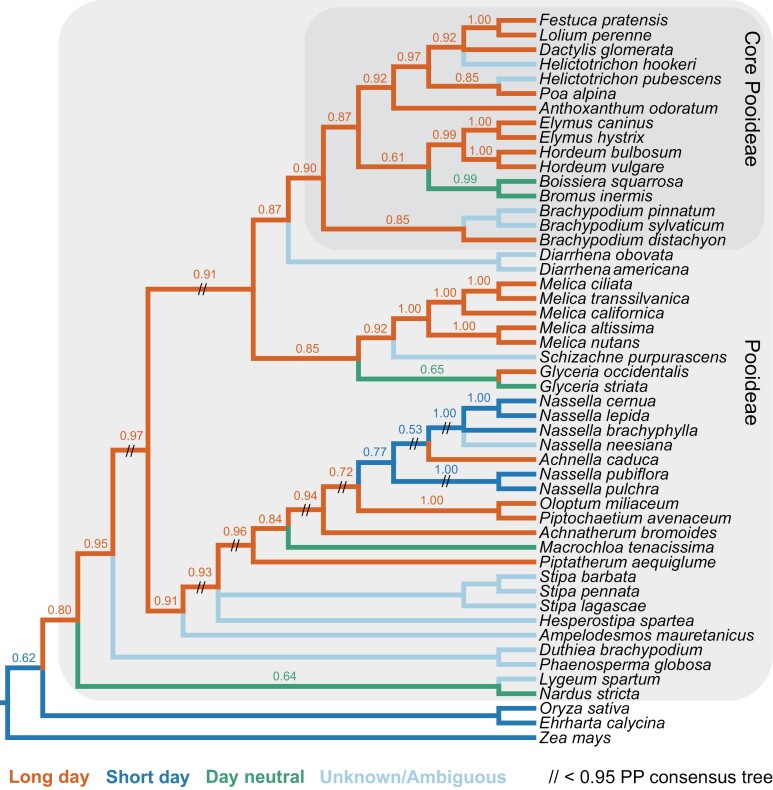
Consensus Bayesian Pooideae tree showing Bayesian state reconstruction for photoperiodicity in flowering. Colored internal branches refer to best-supported [>0.50 posterior probability (PP), shown as numbers above branches] inferred character states: long day (red), short day (dark blue), and day neutral (green). Extant species with light blue branches did not flower, and the internal branches were inferred as ambiguous (PP=0.33 long day, 0.33 short day, and 0.33 day neutral). Tip branches are colored based on results of experiments (see Fig. 1). The topology is supported by >0.95 PP except branches bearing a double backslash. Outgroups are *Zea mays* (Panicoideae), and *Ehrharta calycina* and *Oryza sativa* (Oryzoideae).

To reconstruct the ancestral history of Pooideae photoperiodic flowering, we added several GenBank accessions to our new chloroplast dataset, resulting in alignment lengths of 1582 bp for *matK*, 1348 bp for *ndhF*, and 1104 bp for *rbcL*. Bayesian ancestral state reconstruction based on this concatenated dataset and flowering behaviors supported an early origin of long-day-induced flowering at or around the base of Pooideae (Fig. 2). In addition, at least four transitions to day-neutral flowering were inferred to occur in as many tribes across the tree, and one origin of short-day flowering was inferred near the base of *Nassella* (tribe Stipeae). We here ignore previous reports on day-neutral flowering in artificially selected crop cultivars (Dubcovsky *et al.*, 2006; Beales *et al.*, 2007; Faure *et al.*, 2012; Campoli *et al.*, 2013; Nishida *et al.*, 2013; Turner *et al.*, 2013; Pankin *et al.*, 2014), as we focus on reconstructing the natural evolution of photoperiodic flowering. No transitions from short- to long-day photoperiodic flowering were inferred in Pooideae. The position of long-day *Achnella caduca* within the short-day *Nassella* tribe should be qualified by it being a hybrid between *Nassella viridula* and *Achnatherum hymenoides*. These data support the hypothesis that loss of long-day flowering (i.e. day neutrality) is easier than to gain than short-day flowering, or that there has been stronger selection pressure for the former.

### *FT/VRN3* mRNA is a consistent marker of flowering

To complement our ancestral reconstruction with gene expression analyses, we conducted a second flowering time experiment under different photoperiods in exemplar species: outgroup *E. calycina*, long-day Pooideae *M. ciliata* and *O. miliaceum*, and short-day Pooideae *N. pubiflora*. Unexpectedly, *E. calycina* plants failed to flower under either long or short days in our follow-up experiment. The lack of adult vegetative or inflorescence meristems at day 27 in both photoperiods suggests that these plants failed to become competent to flower (Supplementary Fig. S1). This result was consistent with no detectable *FT/VRN3* expression. 

**Fig. 3.**
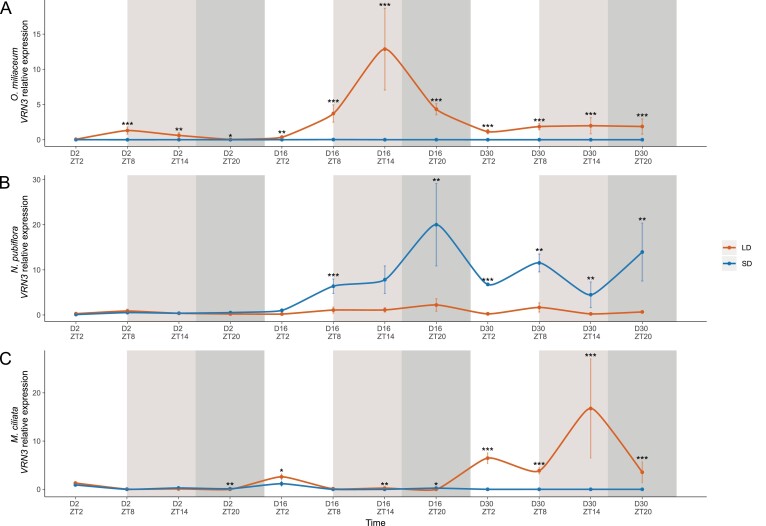
Relative expression of *VRN3* in long- or short-1 day treated plants of (A) *Oloptum miliaceum*, (B) *Nassella pubiflora*, and (C) *Melica ciliata*. Sampling time points are given as Zeitgeber time (ZT) indicating hours after dawn per sampling day. Error bars indicate standard error. White background represents time points that are in the light period in both treatments, light gray background represents time points that are in the dark in the short-day treatment and in the light in the long-day treatment, whereas dark gray background represents time points that are in the dark in both treatments. ***P<0.001, **P<0.01, *P<0.05.

For *M. ciliata* and *O. miliaceum*, ANOVA verified the prediction that *FT/VRN3* expression would be higher in long as compared with short days (*P*<0.001 and *P*<0.001, respectively, Fig. 3), consistent with clear spikelet meristems being visible by day 41 under long but not short days in *O. miliaceum* (Supplementary Fig. S1; data for *M. ciliata* not collected). In contrast, and in line with the observation of well-developed inflorescences at day 41 in short but not long days (Supplementary Fig. S1), *N. pubiflora* showed significantly higher *FT/VRN3* in short days (*P<*0.001) (Fig. 3).

### Pooideae *PHYC* and *PPD1* expression is generally conserved

**Fig. 4.**
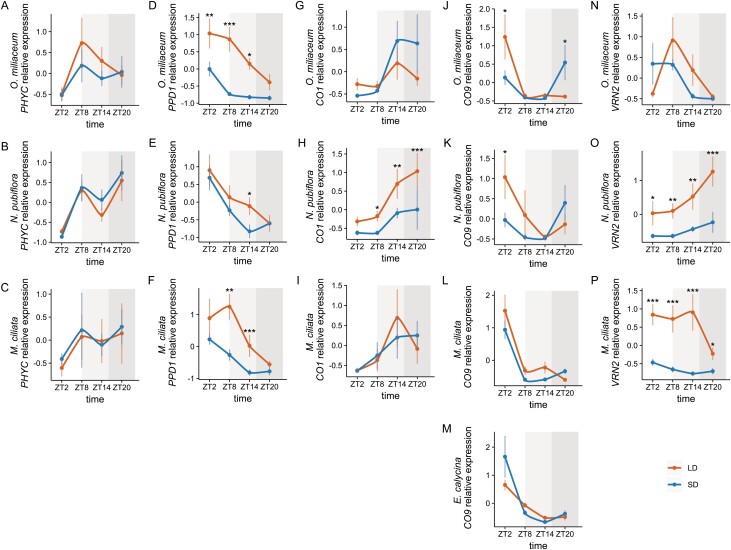
Relative expression of *PHYC*, *PPD1*, *CO1*, *CO9*, and *VRN2* in long- and short-day-treated plants of (A, D, G, J, N) *Oloptum miliaceum*, (B, E, H, K, O) *Nassella pubiflora*, and (C, F, I, L, P) *Melica ciliata.* (M) Relative expression of *CO9* in *Ehrharta calycina*. Sampling time points are given as Zeitgeber time (ZT) indicating hours after dawn. Error bars indicate the SE. A white background represents time points that are in the light period in both treatments, a light gray background represents time points that are in the dark in the short-day treatment and in the light in the long-day treatment, whereas a dark gray background represents time points that are in the dark in both treatments. ***P<0.001, **P<0.01, *P<0.05.

After phylogenetically confirming orthology with other single-copy *PHYC*- and *PPD1*-like grass genes, we determined transcript levels for our focal Pooideae taxa, first to determine any differences between naturally occurring long- and short-day Pooideae, and second to provide context for expression of other CCT genes whose protein products potentially interact with PPD1. For *PHYC*, ANOVA showed no significant effect of photoperiod on expression for long-day *O. miliaceum* and *M. ciliata* or short-day *N. pubiflora* (Fig. 4). In contrast, photoperiod had a significant effect on expression levels of *PPD1* for both *O. miliaceum* and *M. ciliata* (*P*<0.001 for both, Fig. 4). Post-hoc tests showed significantly higher expression in long as compared with short days at ZT2, ZT8, and ZT14 in *O. miliaceum* (*P*<0.005, *P*<0.001, and *P*<0.05, respectively) and ZT8 and ZT14 in *M. ciliata* (*P*<0.005 and *P*<0.001, respectively). ANOVA showed no significant effect of photoperiod on *PPD1* expression in *N. pubiflora* (Fig. 4); however, the post-hoc test showed that expression was higher in long days at ZT14 (*P*<0.05). For all species, expression peaked in the dark in both photoperiods.

### Evolution of *CO1* and *CO9* expression is consistent with derived short-day flowering in Stipeae

Previous authors have suggested that *CO1* and *CO2* were derived from a segmental duplication event at the base of grasses (Higgins *et al.*, 2010). Since both Pooideae copies have been implicated as flowering promoters in the absence of a functional PPD1, or flowering repressors in the presence of PPD1, and *CO1* is expressed more highly than *CO2* at least in wheat, we chose *CO1* for further analysis (Shaw *et al.*, 2020). No effect of photoperiod on *CO1* expression was identified in long-day *O. miliaceum* and *M. ciliata.* However, ANOVA showed a significantly higher expression of *CO1* in long versus short days in *N. pubiflora* (*P*<0.001), and post-hoc tests identified significant differences identified at ZT8, 14, and 20 (*P*=0.05, *P*<0.05, and *P*<0.001, respectively). For all species, expression was at its lowest at ZT2 for both photoperiods and increased throughout the day.

**Fig. 5.**
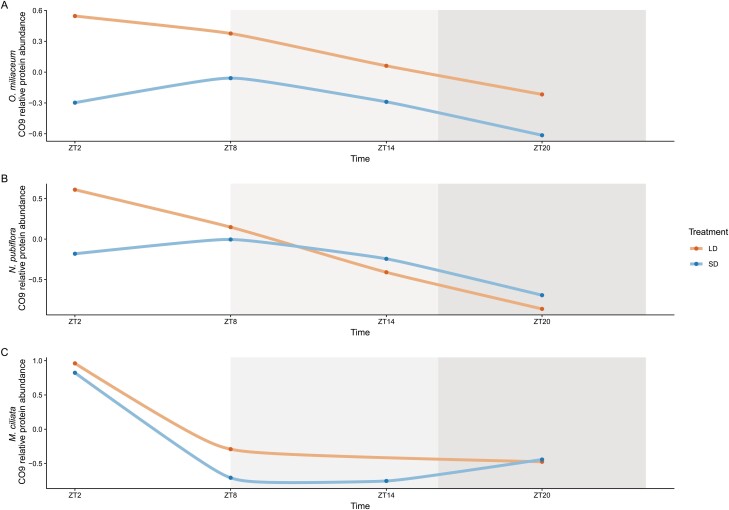
Relative abundance of CO9 protein in long- or short-day-treated plants of (A). *Oloptum miliaceum*, (B) *Nassella pubiflora*, and (C) *Melica ciliata*. Sampling time points are given as Zeitgeber time (ZT) indicating hours after dawn. A white background represents time points that are in the light period in both treatments, a light gray background represents time points that are in the dark in the short-day treatment and in the light in the long-day treatment, whereas a dark gray background represents time points that are in the dark in both treatments.

It was previously reported that barley *CO9* is more highly expressed under short versus long days, and peaks in expression during the light (Kikuchi *et al.*, 2012). No data are currently available for the model species rice or *B. distachyon*. To determine if photoperiod regulation of *CO9* is conserved across the BOP clade, and if changes in regulation are associated with the secondary shift to short-day Pooideae flowering, *CO9* expression was profiled in all focal species (Fig. 4). ANOVA and post-hoc tests showed that *CO9* expression in the short-day outgroup *E. calycina* and long-day *M. ciliata* was similar in abundance across photoperiods, with the peak of expression coinciding with the light period under both conditions (Fig. 4). This pattern for *M. ciliata* CO9 appeared to be confirmed at the protein level based on results of the western blot (Fig. 5; but see the Discussion for potential caveats) (*E. calycina* not tested). ANOVA did not identify a significant effect of photoperiods on expression for *O. miliaceum* and *N. pubiflora CO9*. However, in both species, variation in periodicity resulted in a peak of expression in the light for long days and dark for short days for mRNA (Fig. 4), with a significant difference of expression between long and short days at ZT2 (*P*=0.01, Fig. 4). Furthermore, whereas the peak of mRNA expression was in the light for long days and the dark for short days (Fig. 4), CO9 protein peaked in abundance during the light of both photoperiods and species (Fig. 5), potentially suggesting transcriptional instability or protein degradation in the dark.

### *VRN2* expression has evolved in both long- and short-day Stipeae

*VRN2/Ghd7* is positively regulated by long days in rice and barley (Trevaskis *et al.*, 2006; Xue *et al.*, 2008). To determine if this long-day response is generally conserved, or has evolved in short-day Pooideae taxa, we assessed *VRN2* expression in *M. ciliata*, *O. miliaceum*, and *N. pubiflora* (Fig. 4). Unfortunately, we were unable to amplify the rice *Ghd7* ortholog from *E. calycina*, suggesting either low expression in leaf tissues under our experimental conditions or high levels of sequence divergence relative to rice. ANOVA showed a significant effect of photoperiod on expression of *VRN2/Ghd7* in *M. ciliata* (*P*<0.0001) and expression was higher in long as compared with short days at all time points (*P*<0.01, *P*<0.01, *P*<0.001, and *P*<0.05, respectively, Fig. 4), peaking in the light in both photoperiods (Fig. 4). Contrary to prediction, *O. miliaceum* showed no significant difference in *VRN2/Ghd7* transcript levels between photoperiods (Fig. 4), and expression during the light period in both long and short days. Finally, despite its relatively close relationship to *O. miliaceum*, and its short-day responsiveness, *N. pubiflora VRN2/Ghd7* was expressed at a significantly higher level under long compared with short days (*P*<0.0001) at all time points (*P*<0.05, *P*<0.01, *P*<0.01 and *P*<0.001, respectively, Fig. 4). Interestingly, expression peaked during the dark for both photoperiods (Fig. 4). Beyond expression patterns within species averaged across days, interspecific comparisons of *VRN2* demonstrated relatively weak expression for *M. ciliata* and *O. miliaceum* under both photoperiods, with much stronger expression observed for *N. pubiflora VRN2* under long days by treatment day 16 (Supplementary Fig. S2). Assuming that VRN2 is a conserved repressor of flowering, these data are consistent with stronger long-day suppression of flowering in a short-day versus long-day species.

## Discussion

### Variation in photoperiodic flowering correlates with major niche transitions in the BOP clade

Pooideae is the most dominant grass subfamily of the northern temperate, continental, and Arctic regions (Hartley, 1973). We hypothesized that one of the keys to this success was the use of lengthening days in the spring and summer as a cue to flower rapidly at the appropriate time within limited growing seasons. In line with predictions of this hypothesis, ancestral state reconstruction of photoperiodic flowering responses supports both the dominance of long-day-induced flowering in Pooideae, and its evolution relatively early in the subfamily, after it diverged from Bambusoideae (bamboos). An obvious caveat to our study is the lack of exhaustive sampling across Pooideae, at both the inter- and intraspecific level. However, we believe our attempt to capture accessions spanning geographic variation within the subfamily makes our findings robust to any sampling deficits.

An early origin of vernalization-mediated flowering was previously reconstructed for Pooideae (McKeown *et al.*, 2016), which together with our data (Fig. 2) suggests that the dual photoperiod–temperature induction of flowering long known from winter Pooideae cereals (Heide, 1994) was a key step toward colonizing newly expanding temperate climates. Recent dating of the grasses (Burke *et al.*, 2016; Gallaher *et al.*, 2019; Schubert *et al.*, 2019a) places the origin of Pooideae at the transition between the Cretaceous and Paleocene, 60–70 million years ago (Mya), at a time when mean temperatures were relatively high (Zachos *et al.*, 2001) and seasonality in temperature relatively low (Archibald *et al.*, 2013). Biogeographic studies suggest a Eurasian origin for Pooideae (Bouchenak-Khelladi *et al.*, 2010), and a recent reconstruction of the ancestral niche of Pooideae suggests that its ancestor experienced frost (Schubert *et al.*, 2019a), consistent with a cold micro-habitat origin, possibly in montane Eurasia. Together, these results imply that Pooideae was already to some degree adapted to the cool, seasonal northern climates that developed after the Eocene–Oligocene (E–O) boundary 34 Mya (Strömberg, 2011), and that the early origins of vernalization responsiveness and long-day flowering played crucial roles in the shift of Pooideae from tropical to temperate regions.

Equally as interesting was the evolution of short-day-responsive species within the Stipeae tribe that correlates with a shift back to the tropics (Fig. 2). Specifically, *Nassella pubiflora*, *N. neesiana*, and *N. brachyphylla* are all native to the South American Andes, although *N. neesiana* has been introduced to other parts of the world (www.gbif.org). On the other hand, *N. cernua*, *N. lepida*, and *N. pulchra* are endemic to California. Faster flowering under the short- versus long-day conditions of our experiment seems counter-intuitive to the fact that *N. pulchra* naturally flowers in June and July. However, we previously found that this species also has a strong vernalization response (McKeown *et al.*, 2016). We thus suggest that vernalization responsiveness has adapted *N. cernua*, *N. lepida*, and *N. pulchra* to the northern warm temperate growth cycle by blocking flowering in the shortening days of warm autumns. Whereas long days alone would delay flowering, the coincidence of lengthening days after a winter cold spell allows some physiological release, resulting in eventual flowering in the summer.

In addition to flowering, many traits, such as abscission, dormancy, cold acclimation, senescence, growth, and metabolism, are under the control of photoperiod (Salisbury, 1981). Molecular crosstalk between the networks controlling these traits has the potential to constrain their evolution through antagonistic or adaptive pleiotropy. In our experiment, most species flowered under both long and short days, although it was usually faster or biased in one condition (Fig. 1). This is consistent with data found for other grass species (Preston and Fjellheim, 2020), and suggests that Pooideae have the molecular machinery to flower under both photoperiods. Given this interpretation, other internal or external constraints must be invoked to account for the strong partitioning in geographic space between the Pooideae and other grass subfamilies (Visser *et al.*, 2014). One possible explanation is that competition prohibits the expansion of species with maladapted flowering phenotypes into areas already occupied by species with more favorable flowering responses (Sherry *et al.*, 2007). If long-day-responsive flowering evolved early in Pooideae species inhabiting a cold Eurasian montane micro-niche, it could have given the Pooideae a competitive advantage and been an important facilitator for the group’s rapid expansion into the emerging and expanding temperate biomes that followed the E–O split.

### Conservation of flowering time gene expression across Pooideae

The ability of many grasses to flower under both long and short days, but still be faster flowering under certain photoperiods, underscores the complexity of the flowering time gene network (Shaw *et al.*, 2020; this study). In this regard, understanding what aspects of flowering control are conserved provides important context to determine how the pathways might have changed. In the case of the evolutionary transition to short-day flowering in Stipeae, we noted that expression of *PHYC* and *PPD1* in our exemplar short-day flowering species *N. pubiflora* broadly matched the pattern found for long-day species (Fig. 4).

*PHYC* conveys photoperiod sensitivity to plants, with wild-type alleles promoting flowering in long-day barley, but repressing flowering in short-day rice (Takano *et al.*, 2005; Nishida *et al.*, 2013). These opposing roles are mediated through epistatic interactions with other flowering time genes, as exemplified by the fact that expression of barley *HvPHYC* actually delays flowering in a rice *phyA/phyC* background (Nishida *et al.*, 2013). In our focal species, *M. ciliata*, *O. miliaceum*, and *N. pubiflora*, *PHYC* mRNA levels were similar under both long and short days, and, as in the case of barley, generally peaked after dusk (Nishida *et al.*, 2013).

*PPD1* is a downstream target of PHYC whose exact function is again affected by epistatic interactions with other flowering time genes (Zhang *et al.*, 2019; Shaw *et al.*, 2020). In wheat and barley, *PPD1* is expressed under both long and short days during the light period, but only accelerates flowering under long days or in response to a flash of light during long nights (i.e. short days) (Nishida *et al.*, 2013; Pearce *et al.*, 2017). Although the *PPD1* ortholog *PRR37* delays flowering under long days in its native rice (Zhang *et al.*, 2019), like *PHYC* its expression in long-day plants accelerates flowering, suggesting conservation of protein function (but see the effect of mutant alleles on day-length sensitivity) (Koo *et al.*, 2013; Shaw *et al.*, 2020). *PPD1* transcript abundance in *O. miliaceum*, *N. pubiflora*, and *M. ciliata* peaked in the light in both treatments (Fig. 4), as in wheat and barley (Shaw *et al.*, 2020; Gauley and Boden, 2021), and was higher under long versus short days. Given the roles of *PHYC* and *PPD1* in photoperiodicity, it is not surprising that their expression patterns are conserved across long- and short-day grasses. On the other hand, it would be interesting to assess perturbations in their expression patterns that might explain loss of long-day photoperiodism in non-core Pooideae, such as high latitude *Nardus stricta* and the widespread Eurasian–North American *Glyceria striata*.

### Evolution of *VRN3* and CCT family gene expression in both short- and long-day flowering Pooideae

As expected based on similar work across a range of angiosperms (Andrés and Coupland, 2012), *FT/VRN3* expression tracked the flowering behavior of our focal Pooideae grasses (Fig. 3). Among others, CCT domain-containing genes are known direct regulators of *FT/VRN3* and often function in a photoperiod-dependent manner (Shen *et al.*, 2020). These attributes make them good candidates to explain evolutionary transitions between long-day, short-day, and day-neutral flowering in Pooideae through the differential regulation of *FT/VRN3*.

In the absence of a functional *VRN2/Ghd7* allele, or when *VRN2/Ghd7* transcripts are low, both *CO1* and *PPD1* have been shown to promote the expression of *FT/VRN3* in grasses, leading to the acceleration of flowering (Campoli *et al.*, 2012; Yang *et al.*, 2014; Mulki and von Korff, 2016; Zhang *et al.*, 2017). For long-day *M. ciliata* and *O. miliaceum*, *CO1* was expressed in a similar manner to rice and sorghum in that its expression level was no different in long versus short days (Fig. 4). However, *PPD1* was more highly expressed in long as compared with short days, whereas *VRN2* expression was low under both photoperiods (Fig. 4). Assuming conservation of the model from wheat, barley, and rice, the lack of strong *VRN2* transcription suggests that CO1–PPD1 will work as part of a floral activator complex under long-day conditions, consistent with long-day-regulated flowering in both *M. ciliata* and *O. miliaceum.*

In contrast to *M. ciliata* and *O. miliaceum*, *CO1* and *VRN2* transcripts were both high specifically under long days in the derived short-day flowering species *N. pubiflora* (Fig. 4; Supplementary Fig. S2). In wheat, barley, and rice, high levels of functional VRN2/Ghd7 form a repressor complex with Hd1/CO1 and PRR37/PPD1 (Yang *et al.*, 2014; Mulki and von Korff, 2016; Fujino *et al.*, 2019). Thus, again assuming functional conservation of the CO1–PPD1–VRN2 complex, these data provide at least a partial mechanism for the evolution of short-day flowering in Stipeae, whereby the VRN2–PPD1–CO1 repressor complex is strengthened specifically under long days.

In addition to the CCT domain-containing genes *CO1*, *VRN2*, and *PPD1*, *CO9* has been implicated as a repressor of flowering under both long and short days in barley, but no data are available for rice or sorghum (Kikuchi *et al.*, 2012). Expression data from the short-day rice relative *Ehrharta calycina* and long-day *M. ciliata* revealed a conserved pattern of expression, with no difference between long and short days, and transcript levels peaking in the light under both photoperiods (Fig. 4). In contrast, *CO9* expression peaked in the morning under long days and in the dark under short days for *O. miliaceum* and *N. pubiflora*, revealing a shift in the diurnal rhythm within Stipeae (Fig. 4). Since light is required to stabilize at least *A. thaliana* CO protein (Hayama *et al.*, 2017), we compared mRNA with protein accumulation in all three non-core Pooideae species and found that the short-day dark peak for *O. miliaceum* and *N. pubiflora* CO9 appeared to deteriorate at the protein level. As a result, CO9 protein was higher under long versus short days, representing a second avenue by which the loss of short-day flowering repression could have evolved in *N. pubiflora* (Fig. 5).

A potential caveat to the protein data relates to the fact that the western blot band for the CO9 antibody was ~8 kDa larger than predicted based on the amino acid sequences derived from transcriptomes of the target species (Supplementary Fig. S3). This result might be interpreted as non-specific binding to off-target proteins. However, given that the results were consistent using two independent antibodies that were designed to avoid cross-targeting to other CO-like proteins, and were generally in line with the mRNA expression profiles, we feel this unlikely. Rather, we posit that the larger size indicates ubiquitination of the target CO9 proteins, which is a common mechanism of regulating flowering time proteins (Piñeiro and Jarillo, 2013). In particular, light–dark regulation of *A. thaliana* CO involves its ubiquitination (Liu *et al.*, 2008).

Assuming correct interpretation of the protein data, less clear is the effect of high long-day CO9 protein expression in *O. miliaceum* that flowers more rapidly under long days (Fig. 5). One possible explanation is that short-day flowering evolved early in Stipeae, with the unique gain of short-day-specific *VRN2* expression in *O. miliaceum* resulting in a novel block to flowering under short photoperiods. One argument against this is the fact that *VRN2* levels are relatively low in *O. miliaceum*. Investigation into further Stipeae species and the use of functional approaches will be required to test these alternative hypotheses.

## Conclusions

Daylength is used as a cue to promote or repress the reproductive transition in most plants, and the photoperiod pathway largely shares a common evolutionary basis (Andrés and Coupland, 2012). We have shown that a switch from short- to long-day induction of flowering was probably a major evolutionary innovation allowing Pooideae grasses to establish and diversify within temperate climates. However, whereas transitions to daylength-neutral flowering are common and phylogenetically widespread, reversions to short-day flowering appear relatively difficult and/or uncommon. We suggest that changes in the diurnal and long-term regulation of *CCT* domain genes by photoperiod have been important drivers of ecologically important niche shifts. Together, these data highlight both the complexity and flexibility of flowering time evolution in plants and provide novel hypotheses that can be tested through further sampling and functional analyses.

## Supplementary data

The following supplementary data are available at *JXB* online.

Table S1. Materials used in the study.

Table S2. Primer sequences used in the study.

Dataset S1. *ndhF* alignment.

Dataset S 2. *matK* alignment.

Dataset S 3. *rbcL* alignment.

Dataset S4. *CO9/VRN2* alignment.

Dataset S5. *PPD1* alignment.

Dataset S6. *CO1* alignment.

Dataset S7. *PHYC* alignment.

Dataset S8. CO9/VRN2 alignment.

Fig. S1. Effect of photoperiod on Pooideae meristem development.

Fig. S2. Relative expression of *VRN2.*

Fig. S3. Western blots of CO9.

erac149_suppl_Supplementary_Dataset_S1

erac149_suppl_Supplementary_Dataset_S2

erac149_suppl_Supplementary_Dataset_S3

erac149_suppl_Supplementary_Dataset_S4

erac149_suppl_Supplementary_Dataset_S5

erac149_suppl_Supplementary_Dataset_S6

erac149_suppl_Supplementary_Dataset_S7

erac149_suppl_Supplementary_Dataset_S8

erac149_suppl_Supplementary_Figures_S1-S3_Tables_S1-S2

## Data Availability

All data are available in the supplementary data. Sequences for constructing the phylogeny are available in GenBank under accession numbers OK020205–OK020264.
