# Supplementary material for: Major niche transitions in Pooideae correlate with variation in photoperiodic flowering and evolution of CCT domain genes
Source: J Exp Bot. 2022 Apr 8;73(12):4079–93. doi: 10.1093/jxb/erac149 (PMC9232202; doi:10.1093/jxb/erac149)
Supplement: erac149_suppl_Supplementary_Figures_S1-S3_Tables_S1-S2 [file erac149_suppl_supplementary_figures_s1-s3_tables_s1-s2.pdf]

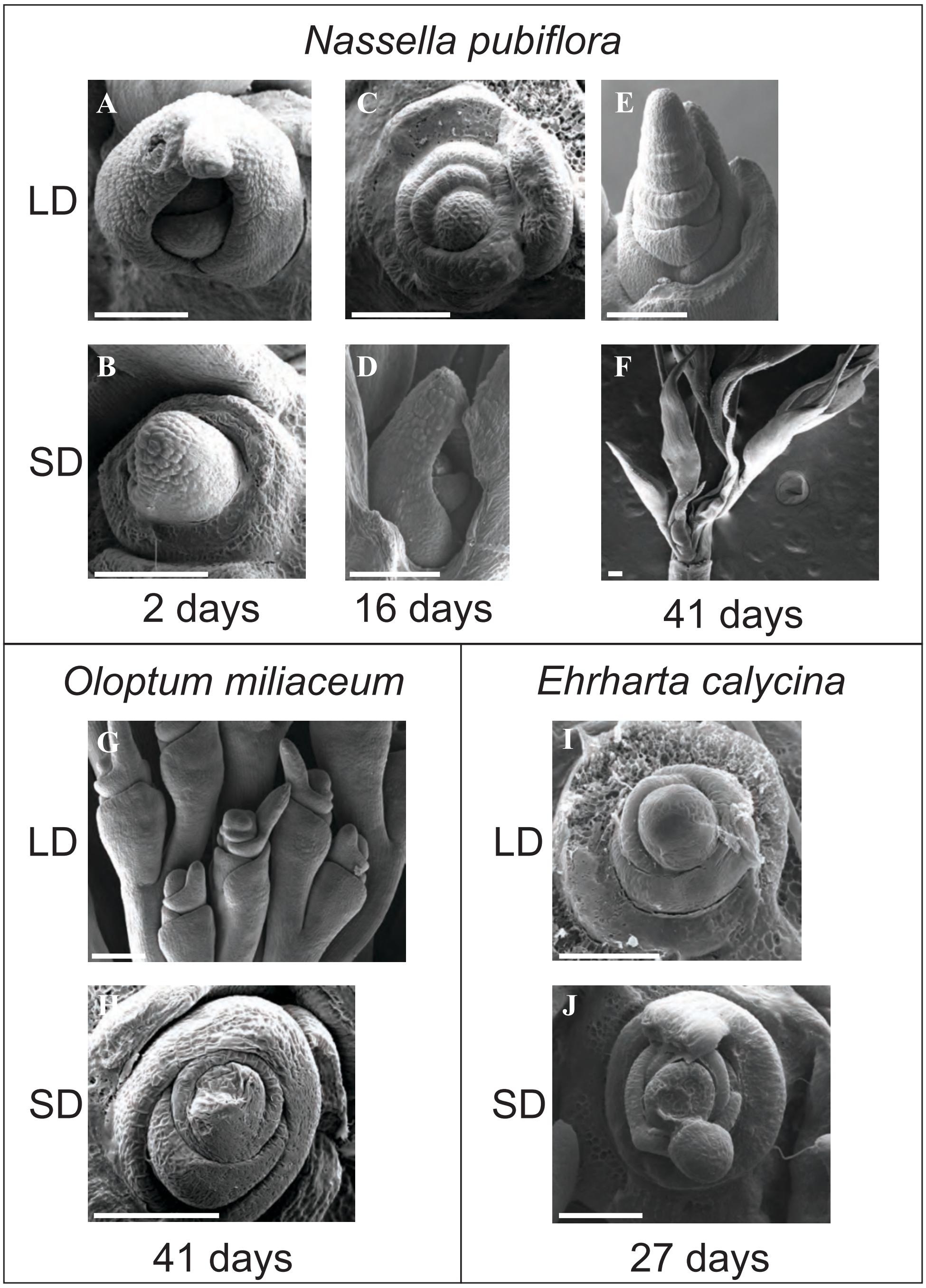

Supplementary figure S1. Effect of photoperiod on Pooideae flowering. A. *Nassella pubiflora* shoot apical meristem (SAM) with 2 long-days. B. *N. pubiflora* SAM with 2 short-days. C. *N. pubiflora* SAM with 16 long-days. D. *N. pubiflora* SAM with 16 short-days. E. *N. pubiflora* SAM with 41 long-days. F. *N. pubiflora* inflorescence with 41 short-days. G. *Oloptum miliaceum* inflorescence with 41 long-days. H. *O. miliaceum* SAM with 41 short-days. I. *Ehrharta calycina* SAM with 27 long-days. J. *E. calycina* SAM with 27 short-days. Scale bar is 100 µm.

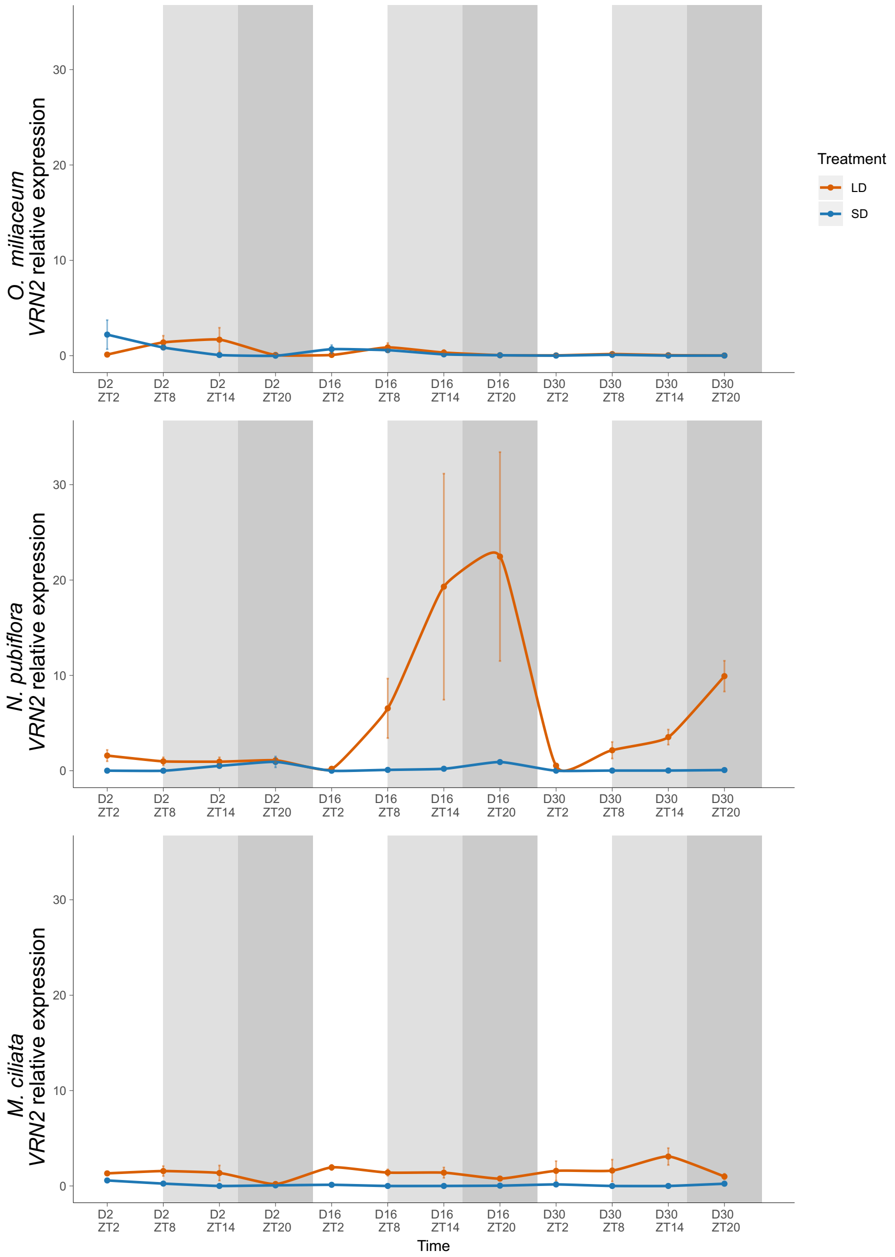

Supplementary figure 2. Relative expression of VRN2 in long- or short-day treated plants of A. Oloptum miliaceum, B. Nassella pubiflora and C. Melica ciliata. Sampling time points are given as zeitgeber time (ZT) indicating hours after dawn per sampling day. Error bars indicate standard error. White background represents time points that are in the light period in both treatments, light gray background represents time points that are in the dark in the short-day treatment and in the light in the long-day treatment, whereas dark gray background represents time points that are in the dark in both treatments

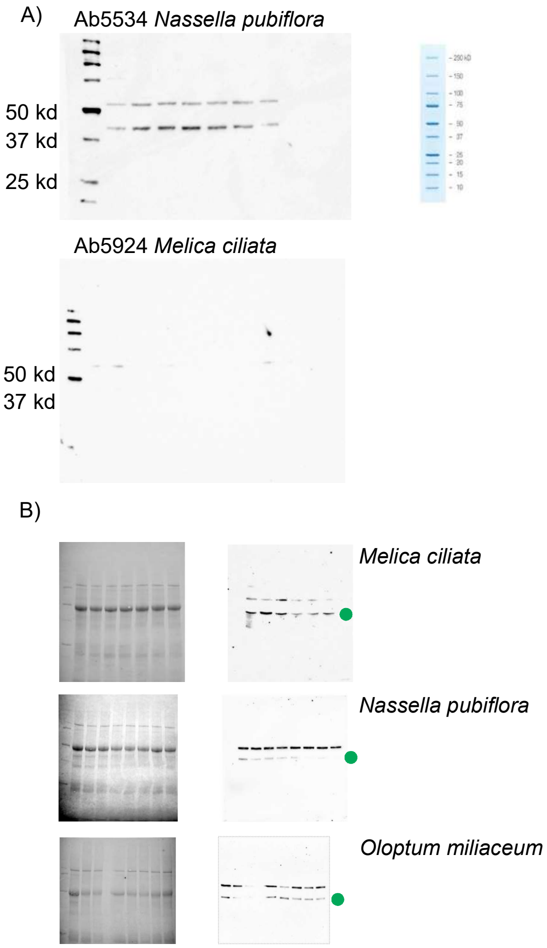

Supplementary figure 3. Western blots of CO9 proteins. A) Size of bands in relation to a ladder for the two antibodies. Staining of the Precision Plus Protein Unstained Standard (Bio - Rad) using StrepTactin-HRP Conjugate was left out in the final blots as the ladder then dominated the blot during exposure B) Exemplary blots for *Melica ciliata, Nassella pubiflora* and *Oloptum miliaceum,* stain free blots to the left and the CO9 blots to the right. Green dots represent the target bands.

Supplemental table 1. Material used in the study.

Species	Seed bank	Accession number	Location
Achnatherum bromoides	GRIN	PI253581	Israel
Achnella caduca	GRIN	PI578861	USA
Anthoxanthum odoratum	NORDGEN	NGB16571	Finland
Ampelodesmos mauretanicus	B&T World Seeds	BTWS 62975	Unknown
Boissiera squarrosa	GRIN	PI314138	Uzbekistan
Brachypodium distachyon	GRIN	PI253334	Morocco
Brachypodium pinnatum	GRIN	PI325216	Russia
Brachypodium sylvaticum		Unknown	Unknown
Bromus inermis	NORDGEN	NGB5420	Norway
Dactylis glomerata	NORDGEN	NGB7723	Norway
Diarrhena obovata	B&T World Seeds	BTWS 516238	Unknown
Diarrhena americana	B&T World Seeds	BTWS 405986	Unknown
Duthiea brachypodium	GRIN	W6 23553, 23539, 23613	China
Elymus caninus	Collected by Thomas Marcussen	TM-Langebåt 2015	Norway
Ehrharta calycina	GRIN	PI284803	Australia
Ehrharta calycina	GRIN	PI578674	USA
Elymus hystrix	MSB	235174	Unknown
Festuca pratensis	NORDGEN	NGB2910	Norway
Glyceria occidentalis	GRIN	Ames31334	USA
Glyceria striata	GRIN	PI387926	Canada
Helictotrichon hookeri	MSB	336026	Canada
Helictotrichon pubescens	MSB	65160	UK
Hesperostipa spartea	GRIN	PI372565	Canada
Hordeum bulbosum	GRIN	PI639320	Tadjikistan
Hordeum vulgare		Cultivar Sonja	
Lolium perenne	NORDGEN	NGB14263	Sweden
Lygeum spartum	MSB	105167	Unknown
Macrochloa tenacissima	GRIN	PI239234	Tunisia
Melica altissima	GRIN	W625184	Kazakhstan
Melica californica	GRIN	W647499	USA
Melica ciliata	GRIN	PI494705	Romania

Melica nutans	GRIN	PI442519	Belgium
Melica transsilvanica	GRIN	PI619447	China
Nardus stricta	Collected by Siri Fjellheim	SF-Røros 2014	Norway
Nassella brachyphylla	GRIN	PI478588	Peru
Nassella cernua	GRIN	W645567	USA
Nassella lepida	GRIN	W645113	USA
Nassella neesiana	GRIN	PI237818	Spain
Nassella pubiflora	GRIN	PI478575	Peru
Nassella pulchra	GRIN	NSL439946	USA
Piptatherum aequiglume	GRIN	PI271588	India
Oloptum miliaceum	GRIN	PI207772	Israel
Piptochaetium avenaceum	GRIN	PI266189	Jordan
Phaenosperma globosum	B&T World Seeds	BTWS 448347	Unknown
Poa alpina	NORDGEN	NGB1197	Sweden
Schizachne purpurascens	MSB	428103	USA
Stipa barbata	GRIN	PI384952	Iran
Stipa lagascae	GRIN	PI252059	Jordan
Stipa pennata	GRIN	PI314395	Russia

Supplemental table 2. Primer sequences used in this study.

Gene	Primer	Sequence	Species	Reference
Chloroplast marker				
ndhF	ndhF_Po_1F	CCGATGCTATGGARGGACCC	Pooideae in Fig. 2	(Schubert *et al.*, 2019)
	ndhF_Po_652F	TTTTTCCCCATAARGATATTGAA	Pooideae in Fig. 2	(Schubert et al., 2019)
matK	matK_Po_1F	TGTTCTGACCATATTGCACTATG	Pooideae in Fig. 2	(Schubert et al., 2019)
	matK_Po_1526	ACGCTCACTGTGTGATCCAC	Pooideae in Fig. 2	(Schubert et al., 2019)
rbcL	rbcL_Po_1F	ACCACAAACAGAAACTAAAGC	Pooideae in Fig. 2	(Schubert et al., 2019)
	rbcL_Po_590R	CATAAATGGTTGTGAGTTTACG	Pooideae in Fig. 2	(Schubert et al., 2019)
Cloning				
VRN2/CO9	CO-like_994f<br>CO-like_1175r	GAGAAGCARATCCGSTAYGMGTC<br>CGGAACCAYCCGAGGTSRAG	NP, OM, EC<br>NP, OM, EC	(Woods *et al.*, 2016)
qPCR				
Ef1α	LolEf1αF	CCTTGCTTGAGGCTCTTGAC	OM, NP, MC	(Woods *et al.*, 2016)
	LolEf1αR	GTTCCAATGCCACCAATCTT	OM, NP, MC	(Woods *et al.*, 2016)
UBQ5	GrassUBQ5F	CGCCGACTACAACATCCAG	NP, OM, EC, MC	(Woods *et al.*, 2016)
	GrassUBQ5R	TCACCTTCTTGTGCTTGTGC	NP, EC, MC	(Woods *et al.*, 2016)
	UBQ5_poace_R1	CAGTAGTGGCGGTCGAAGTG	OM	
Elf4α	Elf4α_poaceae_F2	CGCAAGGTGGACTGGCTCAC	EC	
	Elf4α_poaceae_R2	GAACTCCCTCATGATGATGT	EC	
VRN3	NassPub_VRN3_1012_f	GCAGGAGGTGGTATGCTACG	NP	(McKeown *et al.*, 2016)
	NassPub_VRN3_1304_r	CCCTGGTGTTGAAGTTCTGG	NP	(McKeown *et al.*, 2016)
	OM_VRN3_seq_354F	GGAGGTGATGTGCTACGAGA	OM	
	OM_VRN3_seq_480R	CCTGGTGTTGAAGTTCTGGC	OM	
	cMelica_VRN3_401_f	TGGTCACTGATATCCCTGGAA	MC	
	cMelica_VRN3_612_r	AACAGCACGAACACGAAGC	MC	
	EC_FT_31F	AGCGACCCCAATCTTAGAGAG	EC	
	EC_FT_158R	GTTGAAGTTCTGGCGCCAC	EC	

VRN2	NassPub_qVRN2_f	GGTACGAGTCCAGGAAAGCA	NP	(Woods *et al.*, 2016)
	NassPub_qVRN2_alt.r	GAGGTCGAGTCTGCTTGGATGT	NP	(Woods *et al.*, 2016)
	OM_VRN2a.F3	AGGAAAACTTACGCCGAGATG	OM	
	OM_VRN2a.R3	ACGTCTTGAGCTACCTTGGC	OM	
	MelCil.VRN2.F2	GGAGCCAATTATGGTCATCG	MC	
	MelCil.VRN2.R1	CATGTACCTCGTCACCTTCG	MC	
CO9	NP_CO9_497F	GGAGAGAAATACCGTTCACCG	NP	
	NP_CO9_715R	ACCGGATCTGCTTCTCGTAC	NP	
	OM_CO9_8F	TCTGCGGGAGAGAAACGTTA	OM	
	OM_CO9_241R	ACCGGATCTGCTTCTCGTAC	OM	
	MelCil.CO9.300q.F	CTCGAGCATGTGAAGGGTTG	MC	
	MelCil.CO9.501q.R	AGATGACGGAGAGGTTGCAA	MC	
	EC_CO9_qpcr_188F	GCGTACATAGGCCAAGCATT	EC	
	EC_CO9_qpcr_296R	CTGCTAGTCATCGATCACATACA	EC	
PPD1	OM ppd1 68 F	ACTCGCCATCTCTTCTCCCT	OM	
	OM ppd1 230 R	TTCTTGTGGAGGAAGCGGTC	OM	
	NP ppd1 1601 F	CTGCTCCGATGAAACAGGGT	NP	
	NP ppd1 1790 R	TCACCCATCTTCTTGCCCAC	NP	
	MC PPD1 1212F	GCCGCATGATAACAGCTTGG	MC	
	MC PPD1 1392R	CGCTGACGTGTGTGCATTAG	MC	
CO1	CO1_NPUB_468_F	CAGTGAGAGCAACAACAGCA	NP	
	CO1_NPUB_650_R	ACACACTCGTTCCCTTCCTT	NP	
	CO1_OMIL_414_F	AAAGGAGGTGGAGTCTTGGC	OM	
	CO1_OMIL_645_R	CTCGCTCCCTTCCTTCTCTC	OM	
	MelCil.CO1.FP1	CGTATCAGCAGCAACCAAGAGC	MC	
	MelCil.CO1.RP1	CGCTCAACATTACAGCCTGC	MC	
PHYC	OM PHYC 3867 F	TGGGAGAGCCTAGCTGATGT	OM	
	OM PHYC 3950 R	TCCTGCTCCCCAAACATCAC	OM	
	NP PHYC 592 F	CAGCCTATCAGCCTCTGTGG	NP	
	NP PHYC 720 R	CCCGTCCTCCTCATCCTCAT	NP	
	MelCil.PHYC.FP	CCACTTCGACTACTCCTCGTCG	MC	
	MelCil.PHYC.RP	GCATGTTCTGGAGGTAGGCAGAG	MC	

McKeown M, Schubert M, Marcussen T, Fjellheim S, Preston JC. 2016. Evidence for an Early Origin of Vernalization Responsiveness in Temperate Pooideae Grasses. Plant Physiology **172**, 416-426.
Schubert M, Marcussen T, Meseguer AS, Fjellheim S. 2019. The grass subfamily Pooideae: Cretaceous–Palaeocene origin and climate-driven Cenozoic diversification. Global Ecology and Biogeography **28**, 1168-1182.
Woods D, Mckeown M, Dong Y, Preston JC, Amasino RM. 2016. Evolution of *VRN2/GhD7*-like genes in vernalization-mediated repression of grass flowering. Plant Physiology **170**, 2124–2135.
